# Highly Stretchable PPy/PDMS Strain Sensors Fabricated with Multi-Step Oxygen Plasma Treatment

**DOI:** 10.3390/polym15071714

**Published:** 2023-03-30

**Authors:** Waqar Muhammad, Sam-Dong Kim

**Affiliations:** Division of Electronics and Electrical Engineering, Dongguk University, Seoul 100-175, Republic of Korea; waqar6414@dgu.ac.kr

**Keywords:** strain sensor, polypyrrole/polydimethylsiloxane, oxygen plasma treatment, gauge factor, durability

## Abstract

We present highly stretchable polypyrrole (PPy)/polydimethylsiloxane strain sensors of highly improved sensitivity and durability fabricated by a chemical oxidative polymerization with oxygen plasma treatment (O_2_ PT). In this study, O_2_ PT was performed for 30, 60, and 90 s at each growth stage of the PPy film in three steps to investigate the effects on the sensor performance as well as the microstructural properties of the PPy films. Bonding characteristics with underlying layers and resistance to microcrack generation of the multi-layer PPy films under our given strained state were significantly enhanced by the O_2_ PT. The best sensor performance in terms of sensitivity and stability were achieved by PT for 30 s with a maximum gauge factor of ~438 at a uniaxial strain of 50%, excellent durability over 500 stretching/release cycles, and a fast response time of ~50 ms.

## 1. Introduction

Stretchable strain sensors have gained great recognition in terms of their potential in applications such as continuous health monitoring [1], human body motions [2,3,4], human-to-machine interfaces [5], and intelligent robots [6] with significant research advances in wearable technology. In general, they can be classified into various categories of capacitive [7], resistive [8,9], triboelectric [10,11], and piezoelectric [12,13] sensors depending on their operation mechanism. Among all of these sensing devices, the resistive strain sensors have been the most preferred and intensively studied because of their simple structure, user-friendly and secure signal acquisition, and high sensing stability [14,15,16]. Nonetheless, it is still a great challenge to successfully fabricate resistive strain sensors capable of enduring a wide strain range (greater than few tens in percentage), high gauge factor, long-term durability, and good interoperability with body skin.

To meet all these demanding conditions, many different stretchable conducting polymer materials of polypyrrole (PPy) [17,18,19], polyaniline (PANI) [20,21,22], and poly(3,4-ethylenedioxythiophene):poly(styrenesulfonate) (PEDOT:PSS) [23,24] have been intensively studied for their easy integration to flexible and human-skin friendly substrates such as polydimethylsiloxane (PDMS), thermoplastic polyurethane (TPU), and Ecoflex^TM^ rubbers [25,26,27]. More specifically, these conducting polymers, which have recently started to be widely examined as strain sensor films, are subjected to respond to mechanical deformations which, in turn, alters their electrical characteristics, such as change in resistance, because of their reproducibility and stretchability. In particular, strain sensors based on these stretchable conducting polymers have great importance in the everyday health requirements of people which include the tracking of real-time fitness, routine health monitoring, and, more importantly, the diagnostics of early diseases [28]. Among all the properties of stretchable conducting polymers films for strain sensors, stretchability, conductivity, and sensitivity are three fundamental factors which have decisive roles in the performance of the future strain sensors [29]. Due to easy synthesis, low cost in fabrication, stability in oxidized form, high electrical conductivity, and good redox properties, the PPy has been, by far, the most highlighted among the numerous conducting polymers [30,31,32]; however, as a conjugated conducting polymer, the brittleness of PPy limits its practical uses. For this reason, either blending methods with some other polymers or forming the PPy copolymers could, in part, improve the mechanical properties and fatigue endurance of this material [33,34,35].

There have been reports on various PPy-based strain sensors formed on elastomers with excellent elastic properties, and, as one of examples, Niu et al. demonstrated a strain sensor by depositing a PPy thin layer on a fibrous PDMS membrane [36]. This sensor showed distinctive responses and good conductivity at different strain ranges and demonstrated its unique ‘on-off’ mode characteristics of PPy film in monitoring the bending of different joints. Wang et al. also reported multifunctional and highly flexible three-dimensional strain sensors using PPy-coated nickel foam through simple electro-deposition method, and they revealed the unique interior structure and excellent robustness under different stretching conditions as well as very good elastic characteristics [37]. On the other hand, Mufang et al. showed a successful fabrication of PPy/polyurethane (PU) strain sensors demonstrating the high sensitivity and stretchability required for human motion detection [38]. There have been many other reports for conductive PPy/elastomers composites by polymerizing the elastomer/oxidant with pyrrole vapors; however, most of them exhibited a problem of “peel off” during heavy cyclic stress tests with degradation in performance and sensing life-time because of the inherent stiffness and rigidity of PPy films.

In this work, we present a fabrication strategy of strain sensors with highly conducting multi-step grown PPy films strongly anchored on the surface of underlying layers via simple chemical oxidation polymerization plus an oxygen plasma treatment (O_2_ PT) process. To investigate the effects of our synthesis method on the resistance force to crack generation upon cyclic stress conditions, we performed O_2_ PT for various time intervals on each layer of multi-step grown PPy films. Surface morphology and electrical characteristics of the fabricated multi-layer PPy/PDMS strain sensors were also investigated according to different PT conditions under various stress conditions. To examine the sensitivity of our PPy/PDMS strain sensors to tension or bending strain in a large stretching range, two typical human body motions including both subtle and large-scale movements were tested by attaching the sensors to human body parts.

## 2. Materials and Methods

The pyrrole (C₄H₅N, 99.5% in purity) and iron chloride (FeCl_3_, 97% in purity) were from Sigma-Aldrich, South Korea and used without any further purification. N-(3-trimethoxysilylpropyl) pyrrole (C_10_H_19_NO_3_Si, 95% in purity), abbreviated as Py-Silane hereafter, was from ABCR GmbH, Karlsruhe, Germany. The SYLGARD^TM^ 184 kit for silicone elastomer base and curing agent were purchased from Dow Corning Corporation (Auburn, MI, USA). Colloidal silver paste (PELCO^®^, particle size 0.4–1.0 μm) purchased from Ted Pella, Inc. (Redding, CA, USA) was used for the electrode formation of sensor devices at low temperature. First, thin PDMS elastomer substrates were prepared by mixing the silicone elastomer base and curing agent in a volume ratio of 10:1. The liquid mixture was then placed on a watch glass and, after degassing, transferred to a silicon wafer by spin-coating at 200 rpm for 30 s. The spin-coated PDMS was finally cured at 100 °C for 2 h to achieve a final thickness of 0.45 mm and tailored to a rectangular shape (8 × 10 mm) using a surgical blade for further use.

**Figure 1:**
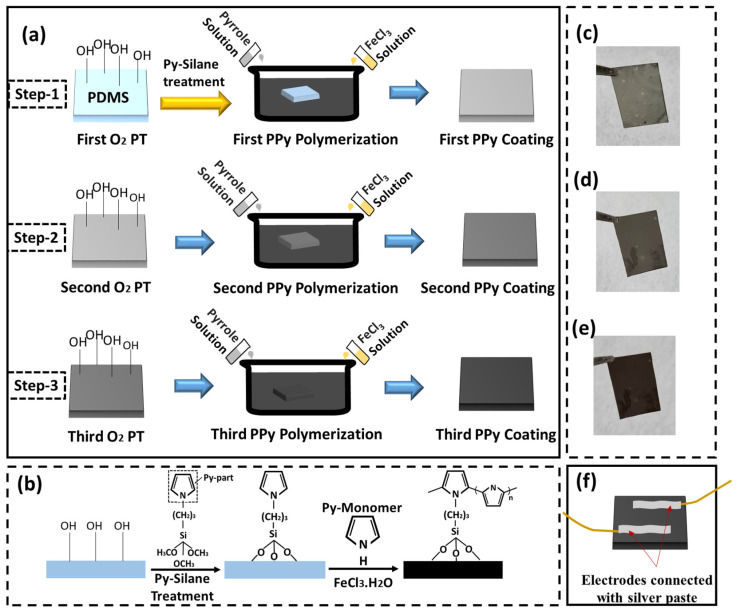
(**a**) Schematic illustrations of the fabrication procedure with O_2_ PT for multi-layer PPy/PDMS strain sensors and (**b**) molecular interactions between PPy and PDMS during the film growth. Photographs of the PPy film surfaces after (**c**) first, (**d**) second, and (**e**) third coatings on PDMS substrate and (**f**) the fabricated PPy/PDMS strain sensor with pasted electrodes.

Schematics of Figure 1a,b describe a chemical oxidation polymerization procedure with three-step O_2_ PT to fabricate our multi-layer PPy/PDMS strain sensor structures. In the first step, the PDMS substrate was plasma-treated with O_2_ in a reactive ion etching system for 3 min at a RF power of 50 W, O_2_ flow rate of 100 sccm, and a process pressure of 50 mtorr to improve the PPy adhesion to the substrate by enhancing the hydrophilicity. The contact angle (CA) was ~103.7° before PT, and it became significantly smaller (~29.3°) after PT on the PDMS surface. CA measurements were done by a tensiometer (DAS100, Kruss, Germany) with an optical goniometer using the water sessile drop method. As illustrated in Figure 1, this O_2_ PT resulted in the formation of highly populated silanol groups (Si-OH) on the PDMS substrate and lead to the substrate surface being more reactive to the silane groups such as Py-Silane, used for a seed layer in this study. The PDMS substrate was then placed in a vial glass with a drop of ~5 μL Py-Silane near to it, and the setup was kept in a vacuum oven and heated at 70 °C for 3 h. During this vacuum vapor treatment of PDMS surface by Py-Silane, the silane components of the Py-Silane monolayer are to be coupled with the hydroxyls (-OH) on PDMS as shown in Figure 1b. Our chemical oxidation polymerization process was carried out using FeCl_3_ as an oxidant, and it lead to the pyrrole monomers being subjected to surface-initiated polymerization. PPy polymerization was carried out by dipping the O_2_ plasma-treated PDMS substrates into the growth solution mixture (for 30 min) which was prepared by mixing the aqueous solutions of FeCl_3_ (0.5 M/12 mL) and pyrrole (1 M/12 mL). During the polymerization process, an interaction between the Py part (see Figure 1b) of Py-Silane with Py-monomer takes place, and it creates the strong covalent bonds of PPy on the PDMS surface as shown in Figure 1b. After the first-step growth of PPy films, the samples were taken out from the growth solution and rinsed in deionized water with ultrasonic agitation (for 3–4 min) to remove any loosely coupled polymers on the surface.

The PPy layer grown on PDMS by the first step is shown in a photograph of Figure 1c. Before the second-step growth, the films grown in the first step were plasma-treated with O_2_ as shown in the second row of Figure 1a. When the PPy films are grown by this multi-step method with O_2_ PT, oxygen radicals present in the vacuum play an important role in changing the PPy micro-fibers to a stronger and more densely cross-linked structure bound more tightly to the underlying layers. After the second-step growth in the growth solution for 30 min as shown in Figure 1d, third-step growth was performed in the same way as illustrated in the third row of Figure 1a. The third layer of PPy film synthesized in this step is shown in a photograph of Figure 1e. A lower RF power of 15 W but the same O_2_ flow rate (100 sccm) and process pressure (50 mtorr) were used for the O_2_ PT in the second and third steps to minimize the plasma damage on the PPy films. Multi-layer PPy/PDMS strain sensors with three different PT time intervals of 30, 60, and 90 s were fabricated, where the control sample (CS) with no treatment (PT for 0 s) was also prepared to investigate the effect of oxygen plasma exposure to the sensor performance. The PPy films synthesized in this procedure showed the total thickness in a range of 500 ± 30 nm as measured by cross-sectional SEM. Electrodes for each sensor element were finally formed using silver paste at room temperature and wired for further measurement as shown in Figure 1f.

Microstructural properties of PPy films on PDMS prepared under different O_2_ PT conditions were characterized by a field emission scanning electron microscope (FESEM, S-4800, Hitachi, Japan) at 15 kV and optical microscopy (BH3-WHP6, Japan). Electrical resistances of the fabricated sensor devices were measured at room temperature through the electrodes formed on the PPy surface using a Keithley 2450 source meter unit. Normalized resistance change (ΔR/R_o_) upon static and cyclic uniaxial stretch/release for the sensor devices was measured by a universal tensile test machine (Digital Tech, Japan), where R_o_ is the unstrained resistance, and ΔR is the resistance change (ΔR = R − R_o_) upon uniaxial stretching. A uniaxial stretch/release test was performed at a strain rate of 100 mm/min, a tensile force of 5 N, and a clamp distance of 10 mm.

## 3. Results and Discussion

### 3.1. Surface Microstructural Morphology

**Figure 2:**
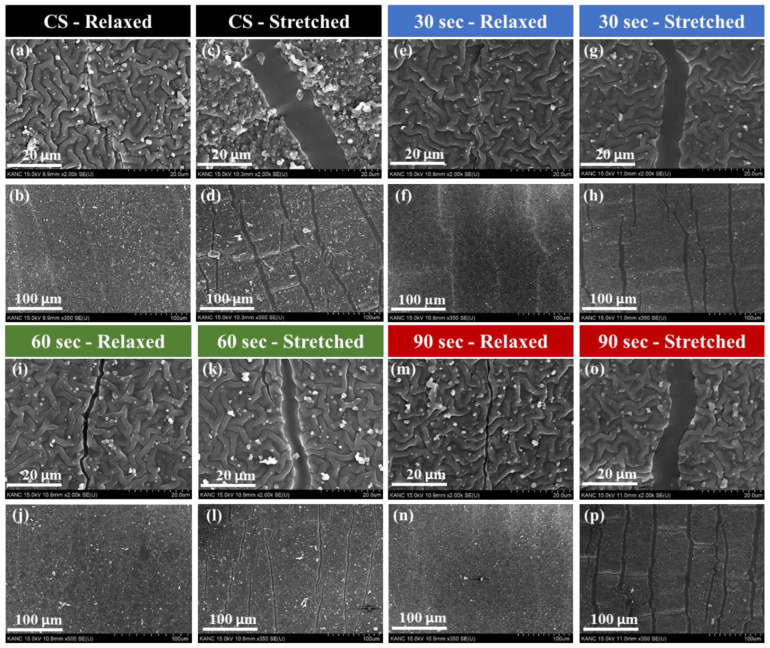
SEM top-views of the PPy films on PDMS substrates synthesized under different PT conditions of (**a**–**d**) CS (no O_2_ PT), (**e**–**h**) 30 s O_2_ PT, (**i**–**l**) 60 s O_2_ PT, and (**m**–**p**) 90 s O_2_ PT. A total of 500 cyclic loads of strain (20%)/relaxation were applied in advance to the PPy films before SEM observation. Images shown on the left columns of each PT condition were taken under relaxed condition at two different magnifications (top: ×2000, bottom: ×350). Images shown on the right columns were taken under the same uniaxial stretching condition (5%) to make the surface microcracks more visible.

A total of 500 cyclic stresses of 20% uniaxial stretching/release were applied to the multi-layer PPy/PDMS structures fabricated under different O_2_ PT conditions before SEM observation. Surface morphologies of the PPy films at two different magnifications (top: ×2k, bottom: ×350) are shown in the SEM top-view images of Figure 2a–p. Micrographs in the left columns for each PT condition were taken under the relaxed state. Since most microcracks produced on the elastic PPy surfaces during the cyclic stress are closed in the relaxed state, an additional 5% stretching was applied to open and make the microcrack more visible during our SEM observation. SEM images taken in this stretching condition are shown in each right column.

One clear difference observed in SEM micrographs is that the PPy layers grown with O_2_ PT exhibit a smoother surface with higher surface coverages as depicted in Figure 2e–p, while the PPy films prepared with no O_2_ PT (control sample, CS) shows rougher surface (Figure 2a–d). This observation suggests that the O_2_ PT significantly affects the bonding characteristics of additional PPys to the lower layers such as the PDMS or underlying PPys during the growth. It, in fact, provides rich hydroxyl groups to the surface of underlying polymer layers as well as more hydrophilic surface, thereby allowing the nucleation of PPys to covalently bond more actively with the underlying layer and ensuring the deposition of denser fiber networks [39]. For example, the earlier studies of O_2_ PT or oxygen plasma immersion ion implantation methods showed that the static water CAs on the polymeric surfaces of PDMS, poly(ε-caprolactone), and PPy were decreased from 114, 136, and 98° to ~25, 0, and 3° by their optimum conditions [40,41,42]. In our case, the CA was decreased from ~123 to ~37° after PT on the PPy surface. Thin PPy films with rougher surface also showed more “peel-off” problems which can lead to increase in electrical resistance and lower responsivity upon applied external strain. On the other hand, uniform surface coverage and the formation of a dense micro-fiber network of PPy achievable by the interlayer O_2_ PT is essential for reliable long-term strain sensing performance.

**Figure 3:**
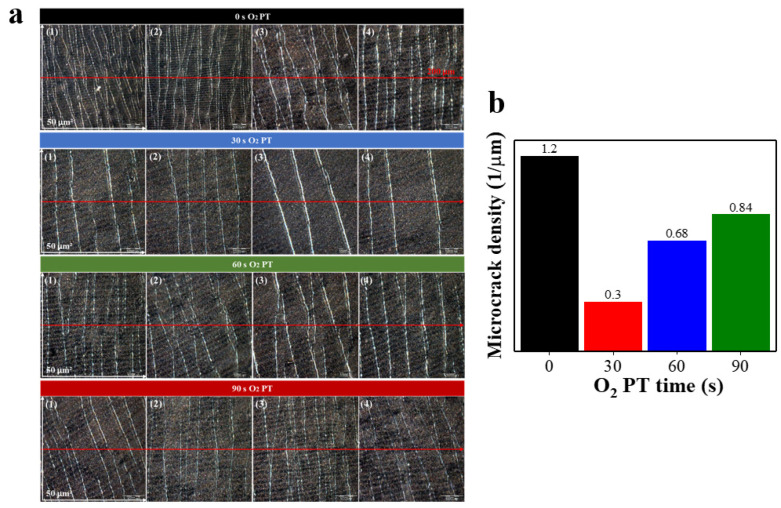
(**a**) Optical microscope top-view images of PPy films synthesized on PDMS substrates under four different PT time intervals of 0 (CS), 30, 60, and 90 s. A total of 500 cyclic loads of stretching (20%)/release were applied in advance to the PPy films before optical microscope observation. Micrographs were taken on four different areas (50 × 50 μm^2^) for each PT condition. (**b**) Bar-graphs illustrating the microcrack densities measured from the PPy films plasma-treated for different time intervals of 0 (CS), 30, 60, and 90 s. All the images were taken under the same uniaxial stretching condition (5%) to make the surface microcracks more visible.

Microcrack densities of the PPy films after being stressed by 500 cyclic uniaxial stretching (20%) and release were measured to quantitatively examine the effect of O_2_ PT. A simple linear density to the elongational direction was utilized in this study because all the microcrack lines were aligned to the perpendicular direction with elongation as shown in Figure 3. Optical micrographs were taken at four different areas (50 × 50 μm^2^) under a sustaining strain of 5% so that all the microcracks could be observed more clearly. Straight lines were drawn to the parallel direction to the elongation for each selected area on optical images, and the average numbers of microcracks intersecting the crossing lines were extracted. Figure 3b illustrates the extracted microcrack densities (number of microcracks per μm) of the multi-layer PPy/PDMS samples prepared under various O_2_ PT conditions. The lowest density was shown in the case of O_2_ PT for 30 s, while the highest was obtained from CS, and this result clearly shows that our O_2_ PT (especially, the maximum effect at PT for 30 s) allows the PPys to grow to a denser fiber network structure, thereby resulting in enhanced resistance to microcrack generation under alternating stress.

### 3.2. Electrical Characterization

**Figure 4:**
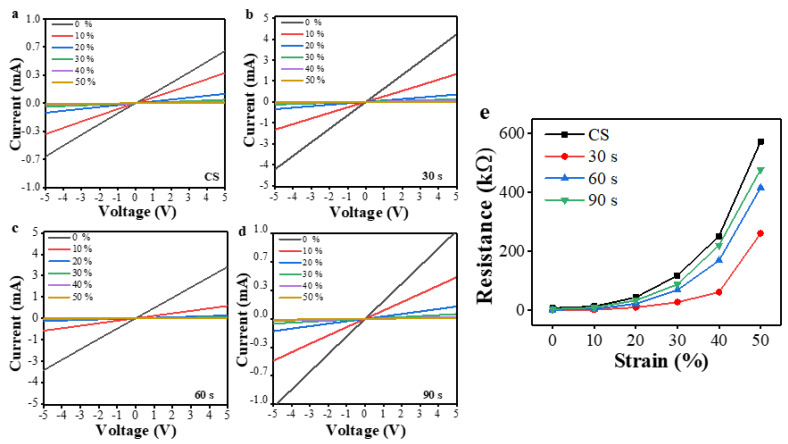
I–V characteristics measured under the axial strain ranging from 0 to 50 % from the multi-layer PPy/PDMS strain sensors fabricated with various O_2_ PT time intervals of (**a**) 0 (CS), (**b**) 30, (**c**) 60, and (**d**) 90 s. (**e**) Electrical resistances measured from the plasma-treated sensors for various time intervals are shown upon the change of film strain from 0 to 50%.

Shown in Figure 4a–d are the typical current-voltage (I–V) characteristics of multi-layer PPy/PDMS strain sensors fabricated using different O_2_ PT conditions (CS, PT for 30, 60, and 90 s) when stressed under various uniaxial strains ranging from 0 to 50% elongation. Regardless of PT condition, all the sensors showed good ohmic properties in a voltage range from −5 to +5 V, but the sensor plasma-treated for 30 s exhibited the lowest electrical resistance (reciprocal of the slope in I–V curve). The electrical resistance progressively increased with the increase of strain in all cases, but the resistances in the case of PT for 30 s were the lowest over the entire range of strain as shown in Figure 4e. As observed in our SEM, the resistance force against microcrack generation of PPy film was maximum at a PT time interval of 30 s but decreased again when the PT was performed for longer time intervals. This phenomenon is not fully understood yet, but it is believed that the increased nucleation rate of PPys caused by higher population of hydroxyls (as well as hydrophilicity) on the surfaces of underlying polymers starts to be saturated to a level of O_2_ plasma exposure time as reported in the previous studies [43]. On the other hand, for PT longer than 30 s, delocalization of the π-conjugated electrons from the backbone chains of PPys can be retarded by the introduction of negatively charged oxygen ions in the plasma state and highly populated hydroxyl groups on the surface; therefore, beyond this point, the intrinsic resistance force of PPy against microcrack generation can start to decrease.

**Figure 5:**
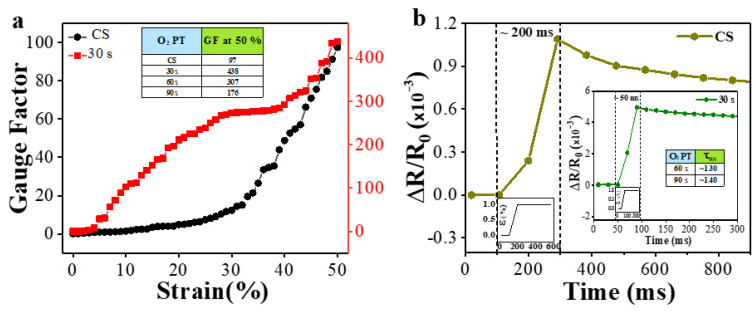
(**a**) GFs (gauge factors) of the multi-layer PPy/PDMS strain sensors fabricated with two different O_2_ PT time intervals of 0 (CS) and 30 s. GFs (at a strain of 50%) of the sensors fabricated with four different O_2_ PT time intervals were summarized in an inset table. (**b**) Transient responses of 0 (CS) and 30 s O_2_ PT PPy/PDMS strain sensors (when a quasi-step function strain varying from 0 to 1% applied as shown in the bottom-left insets of each transient plot) in terms of normalized resistance and an inset table summarizing the transient response times for the strain sensors fabricated with 60 s and 90 s PT conditions.

To further investigate the sensing performance, the gauge factors (GFs) of multi-layer PPy/PDMS structures prepared under different PT conditions were calculated. The GF is defined as (ΔR/Ro)/ɛ where ɛ is the mechanical strain, and ΔR/Ro is the normalized resistance change, and it is an important parameter demonstrating the sensitivity of resistive strain sensors. Plotted in Figure 5 are the calculated GFs as functions of uniaxial strain for the sensors fabricated under two different conditions (CS and PT for 30 s). The maximum GFs of each sensor prepared under every different PT condition at a strain of 50% (ɛ = 0.5) were also summarized in an inset table. As shown in Figure 5a, the CS exhibited the lowest GF of 97 at 50% strain, and the reason behind this low sensitivity is the absence of PT which leads to the loosely bounded PPy layers and the consequent peel-off or break-down under strained condition. On the other hand, the GF values of the sensors prepared with O_2_ PTs showed significant improvement, and, as illustrated in inset table of Figure 5a, the highest GF of 438 was obtained from the case of PT for 30 s (at ɛ = 0.5). Various GFs of stretchable sensor materials on PDMS have been reported such as 2–10 from Ag-nanowire/PDMS and carbon black nanoparticle/PDMS, 137–261 from graphene oxide/PDMS, ~103 from graphene woven fabrics/PDMS, and 3.9–233 from graphene/PDMS composite [44]. In the case of a free-standing silver/PPy composite sensor, a GF of ~21 was reported at a strain of 20% [45]. In addition, the comparison of previously reported flexible strain sensors based on PDMS were summarized in Table 1. The greater GF of a sensor we have, the more easily we can detect small deformations. One notes that a GF of 438 obtained in this study (multi-layer PPy/PDMS structures fabricated with PT for 30 s) at a very high strain of 50% is a significant achievement.

Strain load of a quasi-step function varying between 0 and 1% was applied to the sensors to examine the transient response characteristics. Magnified views of the measured single transient responses from the CS and the sensor plasma-treated for 30 s are, respectively, shown in Figure 5b and additional inset. Response times (τ_RS_) for the CS and plasma-treated (for 30 s) sensors to reach their quasi-steady states were 200 and 50 ms, respectively, while τ_RS_ of the sensors that were plasma-treated for 60 and 90 s were 130 and 140 ms, respectively (see the inset table). As revealed from this measurement, the O_2_ PT for 30 s also brought about significantly reduced response time that can carry out real-time detection and respond to complicated human activities.

**Figure 6:**
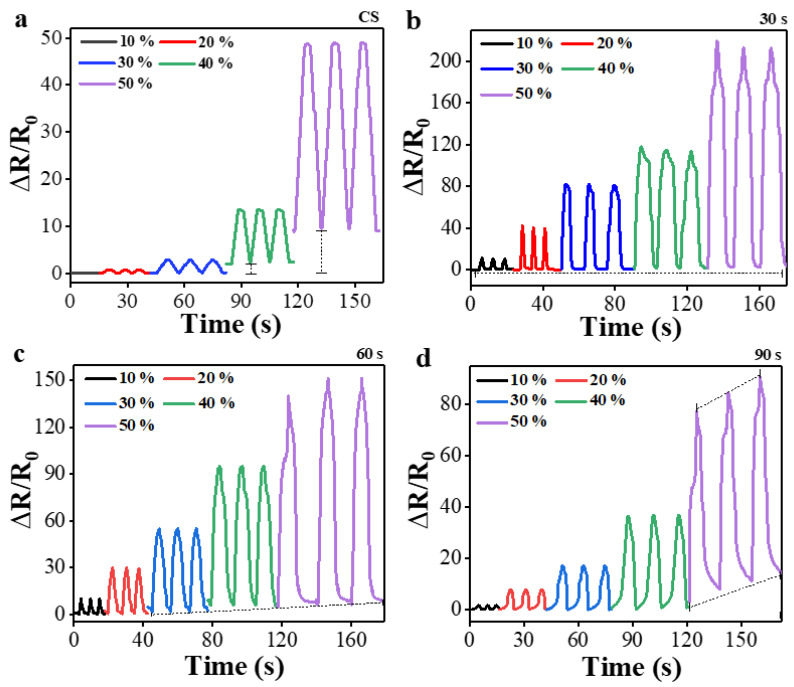
Responses of normalized resistances upon the cyclic strain measured from the PPy/PDMS strain sensors fabricated with various PT time intervals of (**a**) 0 (CS) and (**b**) 30, (**c**) 60, and (**d**) 90 s. In these cyclic tests, the peak strain was varied from 10 to 50% with a stepwise increase of 10%.

To investigate the sensing stability of our strain sensors with the change of strain in magnitude, the responses of ΔR/R_o_ upon the cyclic stretching/release were measured as depicted in Figure 6 by increasing the peak elongational strain from 10 to 50% with a 10% stepwise increase. For these measurements, a constant tensile strain rate of 100 mm/min was maintained. Except for the case of PT for 30 s, the normalized resistances at either peak point (stretching) or valley point (release) measured from all other sensors exhibited unstable changes with the cyclic strain as indicated by dotted lines in the figures. On the other hand, the sensors fabricated with PT for 30 s demonstrated more stable responses under the conditions of both stretching and release with the strain increase of every 10% step (indicated by straight dotted line). It is believed that this stability of normalized resistance is due to the minimizing permanent damage caused by microcrack generation in PPy films through O_2_ PT.

**Figure 7:**
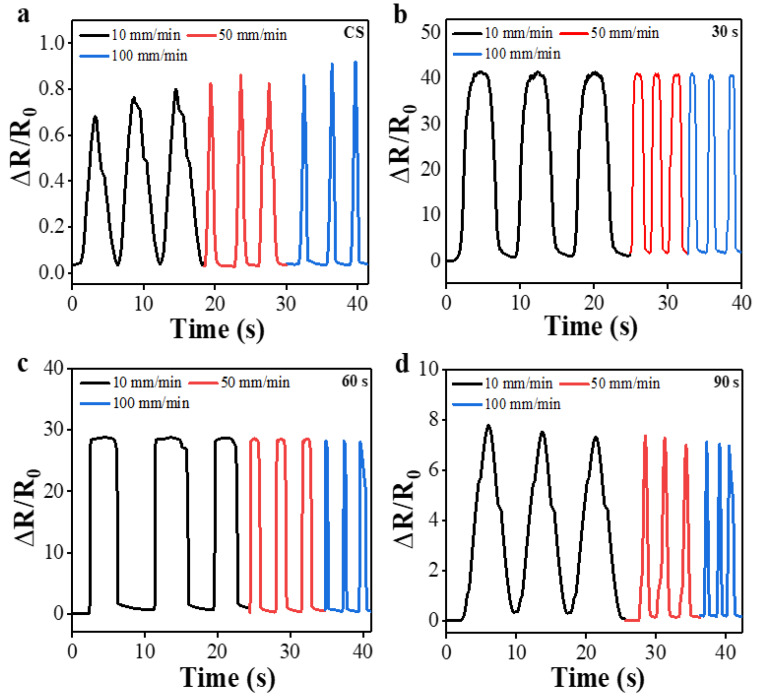
Normalized resistances measured under the different strains rates of 10, 50, and 100 mm/min at a constant strain of 20% from the PPy/PDMS strain sensors fabricated with various PT time intervals of (**a**) 0 (CS) and (**b**) 30, (**c**) 60, and (**d**) 90 s.

To examine the sensing stability depending upon the change of strain rate, the sensors were examined under another stretching/release cycle test condition with three different strain rates of 10, 50, and 100 mm/min after each set of three cycles, as illustrated in Figure 7a–d, but the maximum stretching strain was fixed at 20% in all cases. It was confirmed that the sensor with O_2_ PT for 30 s also exhibited no significant variation in normalized resistance and the stable response at different strain rates as shown in Figure 7b. The CS sensor, however, showed clear and gradual increase in normalized resistance, especially at peak points (stretching), with the increase of strain rate as revealed in Figure 7a. In this test, the sensors prepared with PT for 60 or 90 s exhibited much more stable responses in normalized resistance (see Figure 7c–d) than those of CS.

**Figure 8:**
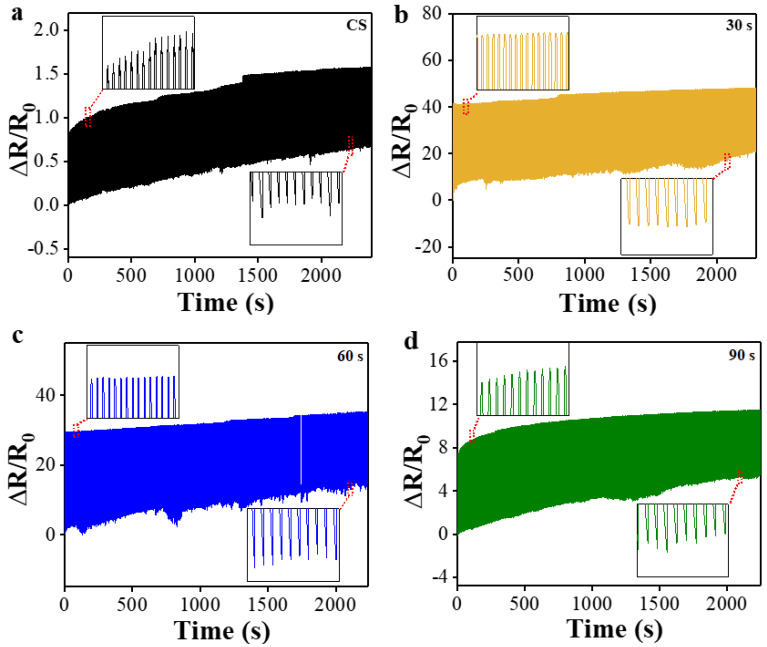
Durability test of the normalized resistance for multilayered PPy/PDMS strain sensors fabricated with various PT time intervals of (**a**) 0 (CS) and, (**b**) 30, (**c**) 60, (**d**) 90 s when subjected to 500 cyclic strains from 0 to 20%.

To assess the durability of multi-layer PPy/PDMS strain sensors, we also performed a mini-marathon test of 500 cyclic stretching/release in real time with a constant peak strain of 20% and a fixed strain rate of 100 mm/min. As shown in Figure 8a, the normalized resistances of CS exhibited significant increase with time at both peak and valley points, which could be attributed to the fatigue fracture and displacement of PPy microfiber structure loosely bounded to the underlying layer due to the absence of interlayered O_2_ PT. On the other hand, the sensors fabricated with O_2_ PT showed more stable peak and valley values and durable performance under this cyclic test as shown in Figure 8b–d. Especially, the sensor with PT for 30 s demonstrated the best performance in durability over the entire range of the cyclic test (Figure 8b).

### 3.3. Sensing Mechanism

**Figure 9:**
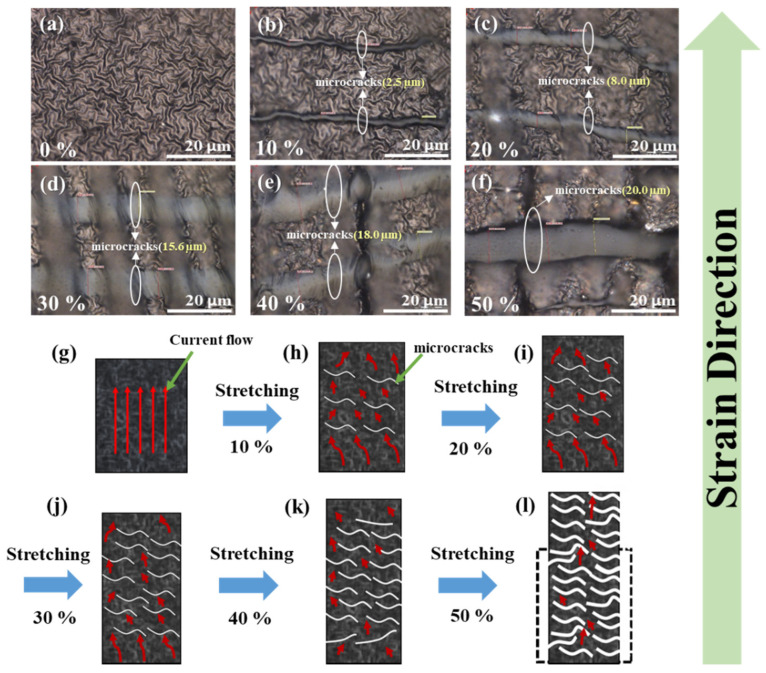
(**a**–**f**) Microstructural surface evolution of the multi-layer PPys on PDMS (O_2_ PT for 30 s) under different tensile strains of 0–50% observed by optical microscopy (×100). (**g**–**l**) Schematics of the PPy film surfaces under different strains where the currents (red arrow) flow bypassing the open circuit paths generated by microcracks.

To understand how our multi-layer PPy/PDMS sensors respond to an external load of stretching, the morphological change on the surface of PPy films (fabricated with O_2_ PT for 30 s) under different levels of elongation (ε = 0, 10, 20, 30, 40, and 50%) were more closely observed using optical microscopy at a ×100 magnification as shown in Figure 9. Under no elongation, PPy multi-layer films obviously show no microcrack and sit stable on the PDMS substrate. When the sensors are stretched to a strain level of 10%, narrow line-shaped microcracks (aligned to the perpendicular direction with elongation) start to appear on the PPy film surface as shown in Figure 9b. Average microcrack line width (w_c_) observed at this strain was 2.5 μm, where w_c_ was measured from four different areas (50 × 50 μm^2^) of each sensor structure. With the increase of strain level, the w_c_ was increased significantly as well as the population of microcracks, and measured w_c_ at 20, 30, 40, and 50% was 8.0, 15.6, 18.0, and 20.0 μm, respectively (see Figure 9c,d).

The formation of microcracks in PPy film structure under a strained state is caused by the random dissipation of strain energy applied to the porous PDMS elastomers, especially by breaking the interfacial adhesion of PPy thin film and random fracturing in the parts of the PPy networked fiber structure; therefore, this process could achieve the effective release of stored strain energy. In the release state, as the PPy/PDMS elastic body is contracted again, the line width of the microcracks generated in the PPy tissue was reduced and even closed. This gave rise to the recovery of many “current paths” and considerable reduction in electrical resistance as illustrated in Figure 9g–l.

### 3.4. Human Motion Monitoring

**Figure 10:**
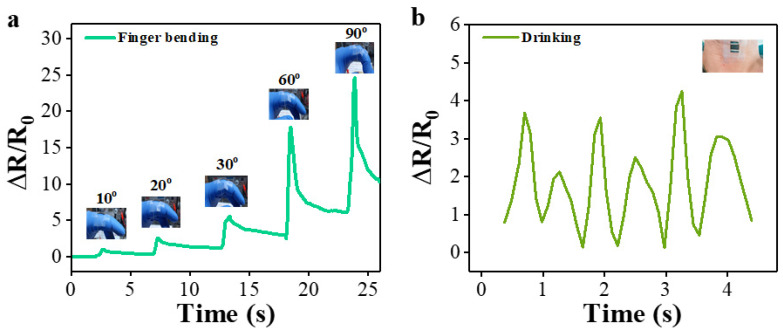
Applications of multi-layer PPy/PDMS strain sensors in monitoring the human body motions. Normalized resistance signals measured from (**a**) the finger’s bending of different degrees (10–90°) to recovery and (**b**) the motion of drinking water.

Due to the electromechanical sensing performance and reliability as discussed above, we were able to examine the feasibility of applying the multi-layer PPy/PDMS sensors fabricated with O_2_ PT to the monitoring of human body movements. Figure 10 demonstrates the change in the resistance signal of the sensor generated by various movements of human-body joints. The strain sensor was first attached to the upper part of an index finger (see Figure 10a), and the corresponding change in normalized resistance was measured according to the finger’s bending movements (bending angle = 10, 30, 45, 60, and 90°). The resistance signals of the sensor exhibited a significant step-like increase as the bending strain changed toward the final bending by 90°. When the finger was fully extended, the resistance immediately returned to its original level. The greater the degree of bending, the higher the relative resistance changed, showing high strain sensitivity (GF of few hundreds) and ultrafast response capability (in few tens of milliseconds). In addition, our PPy/PDMS sensors could effectively monitor subtle human motions such as drinking, swallowing, and chewing. As an example, Figure 10b displays the real time response of resistance upon the very small-scale motion of water drinking when collected from the sensor mounted around a human throat as shown in the inset.

## 4. Conclusions

In conclusion, a chemical oxidative polymerization synthesis method combined with three-step O_2_ PT was demonstrated in this study for the fabrication of highly sensitive and reliable multi-layer PPy/PDMS strain sensor structures. The sensors fabricated with O_2_ PT under an optimized condition showed high sensitivity (GF = 438), a wide sensing range up to 50% elongation, good electrical conductance of PPy films in our sensing strain range of 0–50% (R < 300 kΩ), fast response time (~50 ms), reliable sensing stability at different strain rates, and long-lasting sensing durability upon the cyclic stitching/release. This sensor performance improvement is due to the oxygen plasma exposure to the growing PPy films, which successfully reinforce the bonding characteristics of PPy with underlying layers and provide stronger resistance force to microcrack generation under strained state. Successful extension of this sensor fabrication technology will be applicable to future real-time monitoring of human bodies, flexible robots, and wearable electronic devices and very large and diverse movements.

## Figures and Tables

**Table 1:** Comparison of previously reported flexible strain sensors based on PDMS.

Materials	Methods	Strain Limit (%)	GFs
MWCNTs-PDMS [46]	Liquid Phase Mixing	45	1.2
MWNT-GNPs-PDMS [47]	Printing technique	40	>100
CNT-CB-PDMS [48]	Micromolding method	22.6	29
AgNW-PDMS [49]	Micromolding method	70	2–14
Graphene-PDMS [50]	Coating technique	7.1	2.4–14
PPy-PDMS (our work)	Dipping method	50	438

## Data Availability

Not applicable.
